# Intracardiac injection of a capsid-modified Ad5/35 results in decreased heart toxicity when compared to standard Ad5

**DOI:** 10.1186/1743-422X-9-296

**Published:** 2012-11-29

**Authors:** Raine Toivonen, Juha Koskenvuo, Mari Merentie, Mirva Söderström, Seppo Ylä-Herttuala, Mikko Savontaus

**Affiliations:** 1 Turku Centre for biotechnology, University of Turku, Tykistökatu 6B 5th floor, Turku, FIN-20520, Finland; 2 Department of Medical Biochemistry and Molecular Biology, University of Turku, Turku, Finland; 3 Turku Graduate School of Biomedical Sciences, University of Turku, Turku, Finland; 4 Research Centre of Applied and Preventive Cardiovascular Medicine, University of Turku, Turku, Finland; 5 Department of Clinical Physiology and Nuclear Medicine, Turku University Hospital, Turku, Finland; 6 Department of Biotechnology and Molecular Medicine, A. I. Virtanen Institute for Molecular Sciences, University of Kuopio, Kuopio, Finland; 7 Department of Pathology, University of Turku and Turku University Hospital, Turku, Finland; 8 Department of Medicine, University of Turku and Turku University Hospital, Turku, Finland

**Keywords:** Adenovirus, Intracardiac injection, CAR, CD46, Targeting

## Abstract

**Background:**

Clinical gene therapy trials for cardiovascular diseases have demonstrated the crucial role of efficient gene delivery and transfection technologies in achieving clinically relevant results. We hypothesized that the use of tropism-modified adenoviruses would improve transduction efficacy and to this end we analyzed the transduction efficiency and toxicity of standard Ad5 and tropism-modified Ad5/35 in combination with ultrasound-guided intramyocardial gene delivery.

**Methods:**

Ultrasound-guided intracardiac injections were used to deliver 1 × 10^10^ pfu/ml Ad5-lacZ and Ad5/35-lacZ vectors into mouse left ventricle wall. Since Ad5/35 uses human CD46 as its primary receptor, we used transgenic hCD46Ge mice expressing human CD46 at levels comparable to man. Mice were sacrificed 6 or 14 days post-injection and immunohistochemistry and X-gal staining were used to detect transgene and viral receptor expression. Virus-induced cardiac toxicity was evaluated by a pathologist.

**Results:**

The intramyocardial injection was well tolerated and both Ad5-lacZ and Ad5/35-lacZ were able to give robust transgene expression after a single injection. Interestingly, while Ad5-lacZ was able to generate greater transgene expression than Ad5/35-lacZ, it also evoked more severe tissue damage with large areas of interstitial inflammatory cell infiltration and myocyte necrosis.

**Conclusions:**

Ultrasound-guided intramyocardial injection is an effective and safe way to deliver vectors to the heart. The observed severe tissue damage of Ad5-lacZ greatly undermines the efficient transgene expression and suggests that Ad5/35 capsid modification can result in safer adenoviral vectors for cardiovascular gene therapy, although at the cost of some vector transduction efficacy.

## Introduction

Cardiovascular disease (CVD) is the leading causes of death in the western world, even after steady decline due to improved risk control and effective invasive treatment methods. Annually over 190 000 people die from CVD in the UK alone and the health care costs are over 3.2 billion pounds. As existing methods are clearly inadequate, novel treatment methods, such as gene therapy, are clearly needed.

Adenoviruses (Ads) are the most utilized gene therapy vectors. Over 50 different Ad serotypes, divided in six different subgroups (A-F) have been identified to date [1]. Viruses in different subgroups have varying tissue tropism and recognize different cellular receptors. Coxsackie-adenovirus receptor (CAR) has been identified as the primary attachment receptor for many Ads, including the most utilized serotype 5 (Ad5) [2-4]. Group B Ads have been shown to infect cells in CAR-independent manner and the cellular receptor has been identified as CD46 for most group B viruses [1,5,6]. CD46 is a complement regulatory molecule ubiquitously expressed by all nucleated cells in human body [7]. Recently it was also reported that a subgroup of B adenoviruses use desmoglein 2 receptor for primary attachment [8], but serotype 35 was not included in this group.

Several preclinical gene therapy strategies have been used for cardiovascular disease using different viral vectors and non-viral gene transfer and various modes of administration [9]. Most clinical cardiovascular gene therapy trials have used the standard Ad5 vector and one of the lessons from these trials has been the key role of efficient gene transfection and delivery in achieving clinically meaningful therapeutic efficacy.Thus, it has been clear that Ad vectors must be engineered to enhance their ability to transfect the target tissue. Several methods have been developed to transductionally target Ad vectors including the use of bivalent targeting complexes and engineering genetic alterations to viral capsid proteins [10]. One targeting method which has recently been successfully used in many applications is to use hybrid adenoviruses, which are created by interchanging capsid fibers between serotypes, usually with Ad5 receiving the fiber from another serotype [11]. In this study we used such a hybrid vector, Ad5/35, which has fibers from Group B Ad serotype 35 and thus uses CD46 instead of CAR to gain entry into cells. Previous data demonstrate that Ad5/35 hybrid virus is able to efficiently transduce many cell types that are relatively resistant to Ad5 infection, including hematopoietic stem cells, tumor endothelial cells, and several cancer cell types [12-18]. Previously we have reported an analysis of various Ad receptors in both human dilated cardiomyopathy (DCM) hearts and non-DCM hearts demonstrating significant differences in expression patterns of CAR and CD46, with CAR receptor demonstrating higher expression in both DCM and non-DCM hearts [19].

In addition to vector development, the method of gene delivery plays a crucial role in achieving efficient gene transfer to the heart. Various modes of administration have previously been used to deliver genes to the heart, including intravascular, intracoronary, intramyocardial and intramuscular routes. Ultrasound-guided intramyocardial injection of gene delivery vectors is an attractive and clinically applicable concept to directly infect cardiomyocytes. In this study we employed this method to test the hypothesis that the use of tropism-modified adenoviruses would improve transduction efficacy for cardiovascular applications and to this end evaluated the transductional capability and toxicity of a capsid-modified Ad5/35 adenoviral vector in comparison to standard Ad5 vector in the heart.

## Materials and methods

### Human CD46 transgenic mice

The transgenic mouse strain hCD46Ge was a generous gift from Dr. Ann-Beth Jonsson, Uppsala University, Sweden. These mice were constructed by using yeast artificial chromosome technique as described earlier [20]. In our studies the homozygous strain was used. The CD46 receptor expression in hCD46Ge mice has been analyzed earlier and it has been shown to be ubiquitously expressed, with the heart expressing all four CD46 transcript variants [21]. All animal work was done by trained scientists with permission from the Finnish Laboratory Animal Board (license number: ESAVI-2010-05655/Ym-23).

### Human heart samples

Human heart samples were used for immunohistochemistry of adenoviral receptor expression. Samples were a kind gift from professor Petri Kovanen, Wihuri Institute, Helsinki, Finland. Heart samples were obtained from left ventricles of organ donors with no history of heart disease and whose donated heart could not be used for transplantation. Immediately after collection the tissue was frozen in liquid nitrogen and stored at -70°C. The use of human heart samples as control specimens was approved by the Institutional ethics committee of the Helsinki University Central Hospital, and the investigation conforms to the principles outlined in the Declaration of Helsinki.

### Adenoviral vectors

In this study we used two replication deficient adenoviral vectors, Ad5-lacZ and Ad5/35-lacZ. Both vectors harbor *Escherichia coli* β-galactosidase gene under the control of rous sarcoma virus (RSV) promoter. The viruses were prepared by cotransfecting 293 cells with shuttle plasmid pAd.RSV.LacZ and backbone plasmids pBHG10 (for Ad5.LacZ), or pAdΔΨF35 (for Ad5/35.LacZ) as previously described [22]. pAdΔΨF35 is based on pBHG10 (MicrobixBiosystems Inc., Toronto, Canada) and contains the chimeric Ad5/35 fiber gene instead of Ad5 fiber gene [23]. Viruses were isolated from a single plaque, expanded in 293 cells and purified by double cesium gradient ultracentrifugation. Viral particles (vp) were measured using standard absorbance method and resulted in vp concentrations of 1.9 × 10^12^ for Ad5-lacZ and 3.4 × 10^12^ for Ad5/35-lacZ. The plaque-forming units (pfu) were determined by standard agarose-overlay plaque assay on 293 cells resulting in pfu concentrations 9.3 × 10^10^ pfu/ml for Ad5-lacZ and 2.5 × 10^10^ pfu/ml for Ad5/35-lacZ. The vp/pfu ratios were 20 for Ad5-lacZ and 136 for Ad5/35-lacZ. For the in vivo experiments both viruses were diluted to 1 × 10^10^ pfu/ml, indicating that mice injected with Ad5/35-lacZ received 6.65 times more viral particles than Ad5-lacZ mice.

### Intracardiac injections

All animals received ultrasound-guided injection of Ad5-lacZ (n = 8), Ad5/35-lacZ (n = 9), or PBS (n = 4) into myocardium as previously described [24]. Briefly, the procedure was accomplished under 2.5-3.5% isoflurane anesthesia with the use of a Vevo770 (VisualSonics, Toronto, Canada) equipped with a 30-MHz transducer. After obtaining an optimal parasternal long-axis view, the intramyocardial injection was performed via 30-gauge needle by using micromanipulator. A volume of 10 μl (1 × 10^10^ pfu/ml) was injected in each heart delivering 1 × 10^8^ infectious particles of either Ad5-lacZ or Ad5/35-lacZ vectors. During the procedure, mice were immobilized to a warm plate, anesthetized with isoflurane and their heart rate, respiration and body temperature were monitored. No mice died from the procedure and all were sacrificed 6 or 14 days post injection. The intramyocardial injection technique was also validated using methylene blue dye injection to visualize its distribution at myocardial sections after successful injection (Additional file 1: Figure S1).

### Serum samples

Blood was collected from the submandibular vein at time points 6, 48 and 72 h post injection, into Capiject T-MG serum collection tubes (Terumo, USA). Blood was allowed to clot at least 20 min, before centrifugation. Serum was collected by pipetting, snap frozen in liquid nitrogen, and stored at -20°C before use. Cytokines IL-6 and TNF-α were analyzed with custom Milliplex assay kit (Millipore, USA) according to manufacturer’s instructions.

### Histology, Immunohistochemistry and Image Analysis

Each group of treated animals was divided into two endpoint groups 6 days and 14 days post injection. Mice were sacrificed using CO_2_. Heart, lungs, and liver were collected. Both frozen (for X-gal staining of reporter gene analysis) and paraffin embedded (for histological and toxicity analyses) sections were prepared from the injected mouse hearts. In order to limit the number of animals needed in the experiment, frozen sections for reporter gene analysis were performed only from animals sacrificed at 6 days, whereas paraffin embedded sections for histological and toxicity analyses were performed from animals sacrificed at 6 and 14 days post injection. Sections were stained with standard Hematoxylin / Eosin staining.

Five micrometer sections were stained with Vectastain HRP-kit (Vector laboratories, USA). The sections were fixed onto Superfrost plus slides (O. Kindler GmbH, Germany) (frozen sections with cold acetone). The endogenous peroxidase in frozen sections was inactivated with 0.3% H_2_O_2_. Anti-CD46 primary antibody (HPA016903, Sigma, USA) was used in 2 μg/ml dilution. To evaluate signal from nonspecific antibody binding normal Rabbit IgG sc-2027 (Santa Cruz, USA) was used in 4 μg/ml dilution. Vectastain ABC-reagent was applied on the sections (Vector Laboratories, USA), and receptors were stained with diaminobenzidine (DAB, Sigma-Aldrich, USA) and counterstained with Mayer’s hematoxylin (Sigma-Aldrich, USA). Adenovirus induced lacZ expression was visualized with X-gal staining in frozen sections fixed with cold acetone and stained with 0.2% X-gal (5-bromo-4-chloro-3-indolyl-β-D-galactopyranoside) solution according to supplier’s instructions (Promega, Madison, WI) or by immunostaining with anti-β-galactosidase antibody in paraffin embedded sections.

The stained sections were photographed using a microscope and evaluated for tissue damage by pathologist, who was blinded to the experimental setting. We used a grading system which gave 0-2 points for myocyte damage, 0-2 points for interstitial inflammation and 0-1 points for endocardial involvement resulting in a maximum possible score of 5. The inflammatory response was quantified by calculating inflammatory area using ImageJ software (National Institutes of Health, Maryland, USA). To evaluate the spread of adenovirus-mediated transgene expression, the X-gal stained specific areas were measured by counting stained cells using ImageJ software.

### Tissue DNA extraction and PCR

10 mg of tissue was sectioned from frozen lung and liver of Ad5-lacZ and Ad5/35-lacZ injected mice. DNA was extracted with standard techniques: Tissue was first subjected to proteinase K (400μg/ml) treatment, +50°C o/n. Supernatant was collected after centrifugation 12 000 rpm, 5 min. DNA was extracted from the supernatant twice by phenol:chloroform (1:1) extraction and purified with ethanol precipitation.

Purified DNA was used as a template in standard qualitative PCR with Ad specific primers (forward: CCATTTTCGCGGGAAAACTGA and reverse: AAGCGCCATTCGCCATTC) amplifying RSV promoter area and beginning of β-galactosidase gene carried by both Ad5-lacZ and Ad5/35-lacZ vectors. DNA purified from Ad5-lacZ vector stock was used as positive control.

#### Statistical analysis

The results are expressed as mean ± SD or median [interquartile range]. The Shapiro-Wilk procedure was applied to determine whether data are normally distributed. Independent sample t-test or Mann-Whitney U- test were used for comparing Ad5-lacZ and Ad5/35-lacZ. Changes in cytokine profiles were evaluated using one-way ANOVA. Statistical analysis was performed using SPSS 16 (SSPP Inc, Chicago, IL, USA). P-value less than 0.05 was considered significant.

## Results

### Animal model

**Figure 1:**
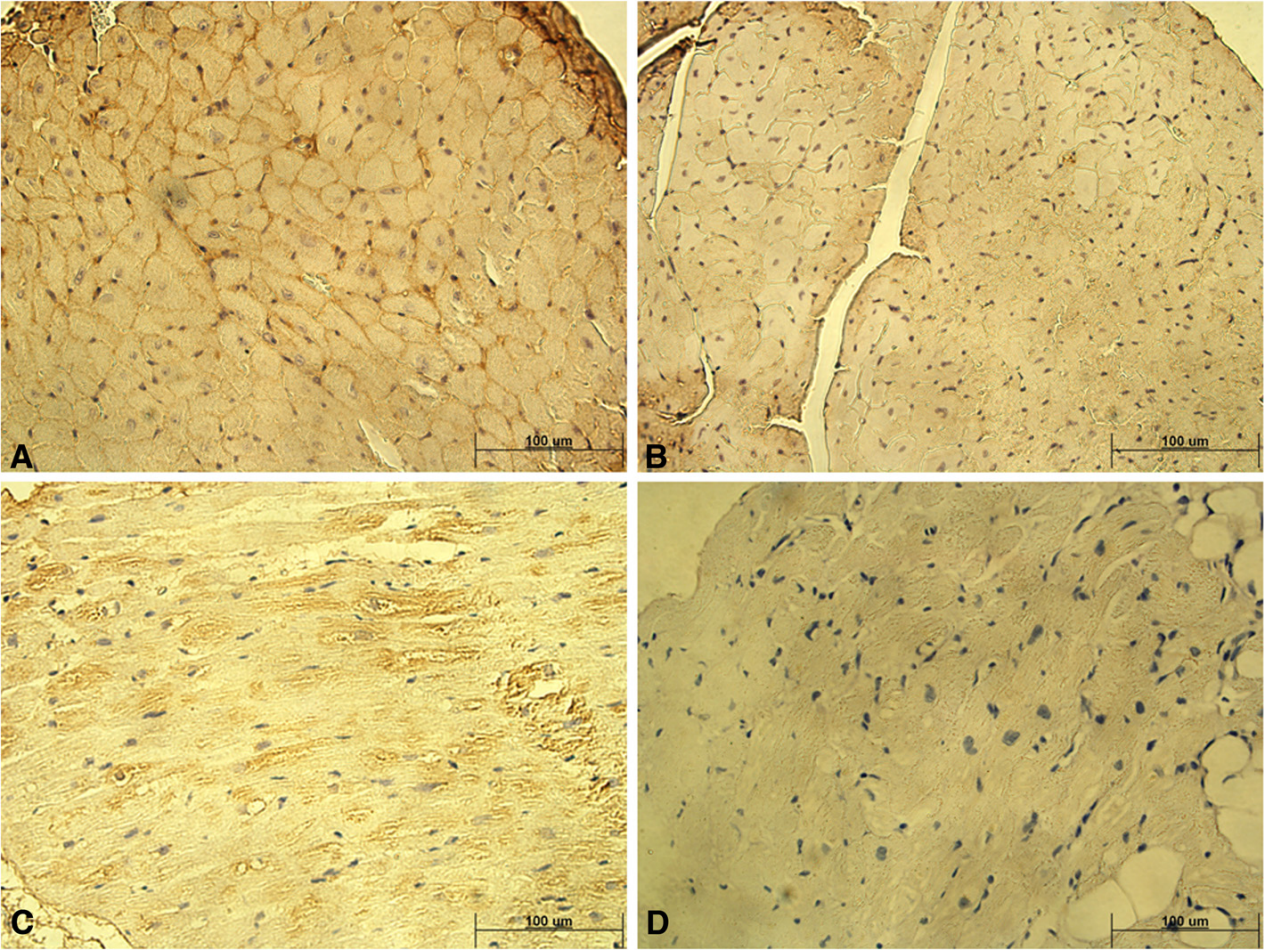
**CD46 expression in the heart. A-B show heart tissue of hCD46ge transgenic mice and C-D human heart tissue.** Panels **A** and **C** show positive anti-human CD46 receptor immunostaining on cell surfaces. Panels **B** and **D** are isotype controls showing the level of background signal. The scale bar represents 100 μm.

As CD46 in mice is different from man and Ad35 uses human CD46 to gain entry into cells, we used transgenic hCD46ge mice, where human CD46 is expressed from a yeast artificial chromosome and has similar CD46 expression pattern as humans. In order to establish the validity of this model we confirmed the receptor expression by immunostaining with anti-huCD46 antibody. Human CD46 expression was found throughout the mouse cardiac tissue at comparable levels to human cardiac tissue, indicating that CD46 expression is comparable in hCD46ge mice and humans (Figure 1).

### Adenoviral transgene expression after intramyocardial injection

**Figure 2:**
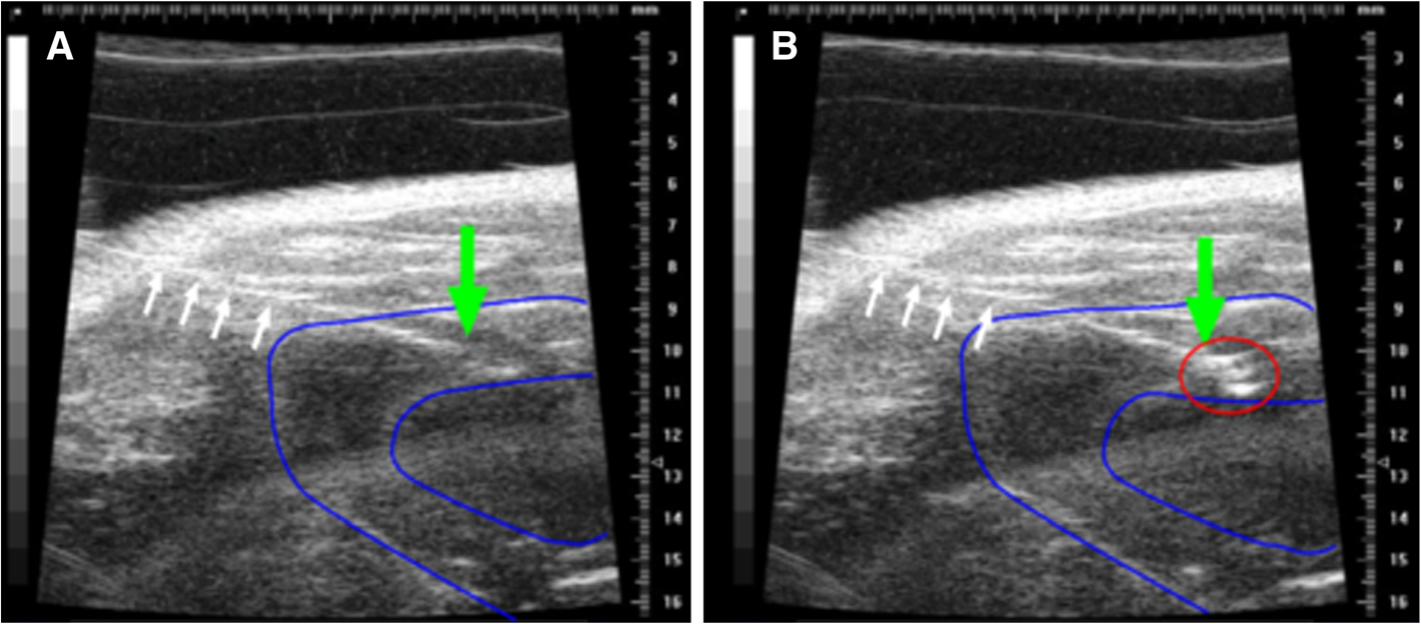
**Successful ultrasound guided injection of left ventricle wall.A**) Needle (white arrows) was inserted in between the ribs into the left ventricle wall (outlined in blue) of anesthetized mouse. The needle tip (green arrow) was positioned intramyocardially without puncturing the left ventricle lumen. **B**) A liquid cloud (red circle) becomes visible and is retained in the ventricle wall after successful injection.

We sought to compare transduction efficiencies between equal amounts of Ad5-lacZ and Ad5/35-lacZ after closed-chest echocardiography guided injection into the free wall of the left ventricle. Echo images show clear liquid cloud during the injection, this cloud is also retained in the heart muscle after successful injection (Figure 2), whereas injection into the ventricle cavity is known to result in rapid clearance of “bubbles” into circulation. We first validated this method by injecting methylene blue to visualize the injected area. The dye was repeatedly retained in the myocardium after successful injection (Additional file 1: Figure S1).

**Figure 3:**
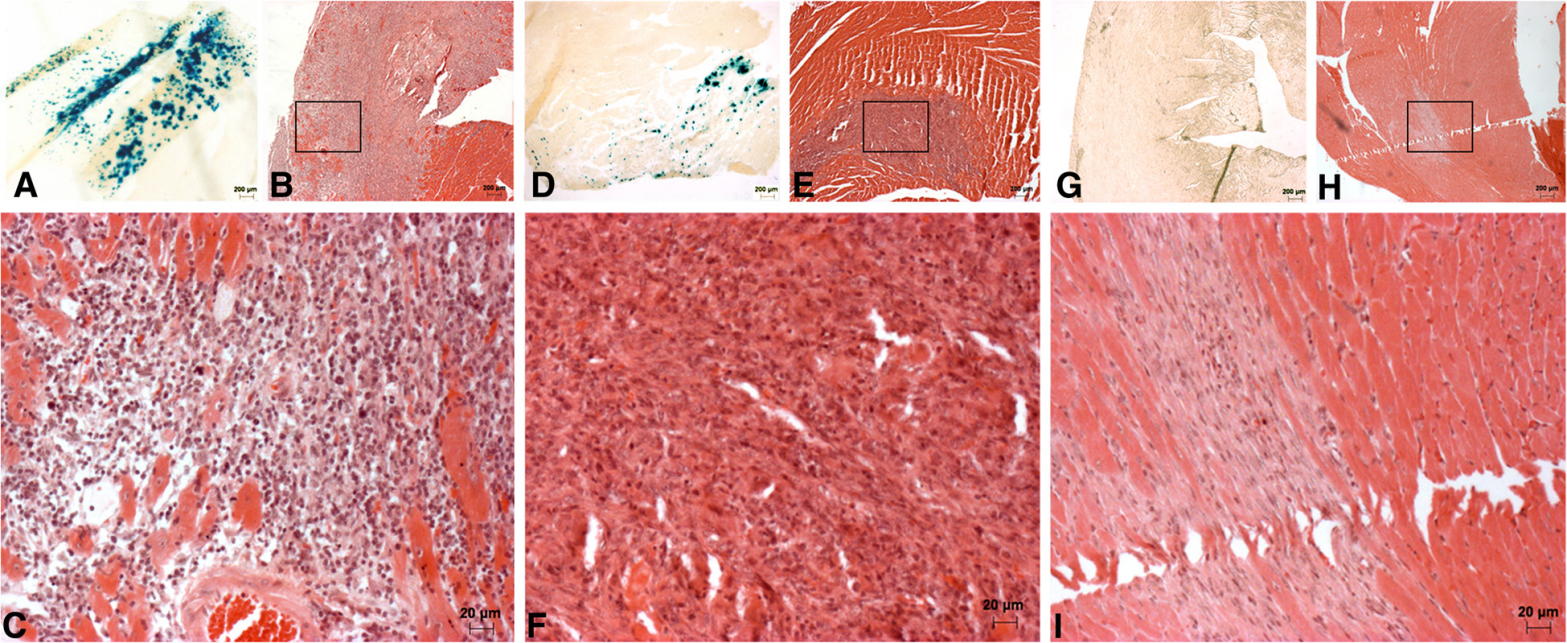
**Transgene expression and inflammatory response after intramural injection of Ad5 and Ad5/35 vectors.** Panels represent transgene expression and tissue damage after injection of Ad5-lacZ injection (**A**-**C**), Ad5/35-lacZ injection (**D**-**F**) or PBS (**G**-**I**). In panel C, a higher magnification image of B shows strong interstitial inflammatory cell infiltration, interstitial fibrosis and myocyte necrosis. Panel F, a higher magnification image of E, reveals weaker inflammation response and absence of myocyte damage. A mild needle tract reaction is seen in PBS-injected hearts (H and I). The sections were HE-stained and panels A, D and G show a light microscopic view from the same myocardial region stained with X-gal to visualize transgene expression. The scale bar in panel F represents 200 μm (for A-B, D-E and G-H) and 25 μm (for C, F and I).

**Figure 4:**
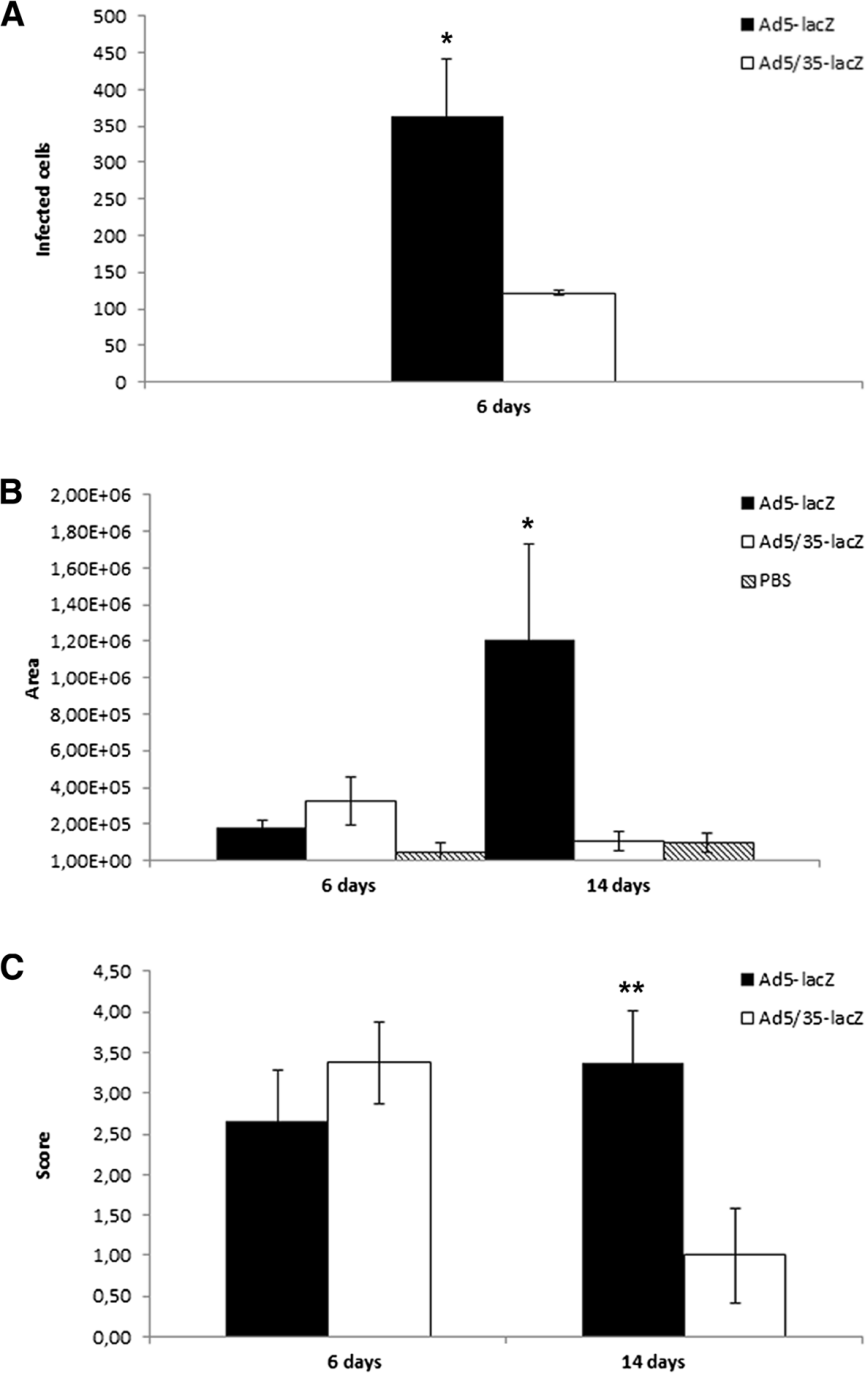
**Quantification of the transgene expression and inflammatory response after intracardiac injection.** ImageJ software was used to **A**) of X-gal positive infected cells at day 6 post injection B) the size of inflammatory response represented here as area A) expression after Ad5-lacZ and Ad5/35-lacZ injection. Ad5-lacZ was able transduce significantly more cells than Ad5/35-lacZ (p = 0.016). **B**) Inflammatory response after Ad5-lacZ and Ad5/35-lacZ injection. Both vectors resulted in mild inflammatory reaction at day 6 post injection. At day 14 Ad5-lacZ was found to be significantly more toxic than Ad5/35-lacZ (p = 0.04). **C**) Grading of the inflammatory response. A pathologist analyzed inflammatory reaction using a grading system for myocyte damage, interstitial inflammation and endocardial involvement. At 14 days the grading score was significantly higher for Ad5 (p=0.009). P-values ** p < 0.01, * p < 0.05.

No mice died from the procedure and no abnormal behavior of animals was observed during the experiment. Expression analyses were performed at 6 days post injection and they revealed that both Ad5-lacZ and Ad5/35-lacZ viruses were able to give robust transgene expression after a single injection of 1 × 10^8^ pfu of virus (Figure 3). The area of transduced cells after Ad5-lacZ injection comprised approximately 13% of the total left ventricle area (Figure 3A).When comparing the transduced areas after Ad5-lacZ and Ad5/35-lacZ injection we observed that Ad5-lacZ was able to transduce 2.99 times more cells as compared to Ad5/35-lacZ(p = 0.016) (Figure 4A). The infected area with Ad5-lacZ was also larger than with Ad5/35-lacZ.

### Toxicity after adenoviral injection

In order to examine toxicity related to the virus injection, the heart and other organs were examined for virus-related toxicity at 6 and 14 days post injection. In the heart there was a clear difference between toxicities elicited by the two viruses. At day 6 post injection both vectors resulted in mild inflammatory reaction. At day 14 Ad5-lacZ was found to be significantly more toxic than Ad5/35-lacZ. Ad5-lacZ vectors induced tissue damage of over 11 times larger area than Ad5/35-lacZ vectors as measured by infiltration of immune cells (Figure 4B). The difference in the size of the affected areas was statistically significant (p = 0.04). Ad5-lacZ showed diffuse and severe inflammatory infiltrate, mainly represented by lymphocytes. In addition myocyte damage and necrosis were clearly seen as well as interstitial fibrosis (Figures 3B and 3C). In comparison Ad5/35-lacZ showed clearly less intense inflammatory infiltrate and no myocyte necrosis (Figures 3E and 3F). At 14 days post injection Ad5/35-lacZ transduced heart sections also showed features of resolving infection with evident interstitial fibrosis. The inflammatory reaction was also quantified by a pathologist with points given for myocyte damage, interstitial inflammation and endocardial involvement with a maximum possible score of 5 (Figure 4C). At 14 days post injection there was a significant difference between the groups with inflammatory grade score 3.4 for Ad5-lacZ and 1.0 for Ad5/35-lacZ (p=0.009). Analysis of viral particle/pfu ratios indicated that Ad5/35-lacZ mice received 6.65 times more viral particles than Ad5-lacZ mice.

**Figure 5:**
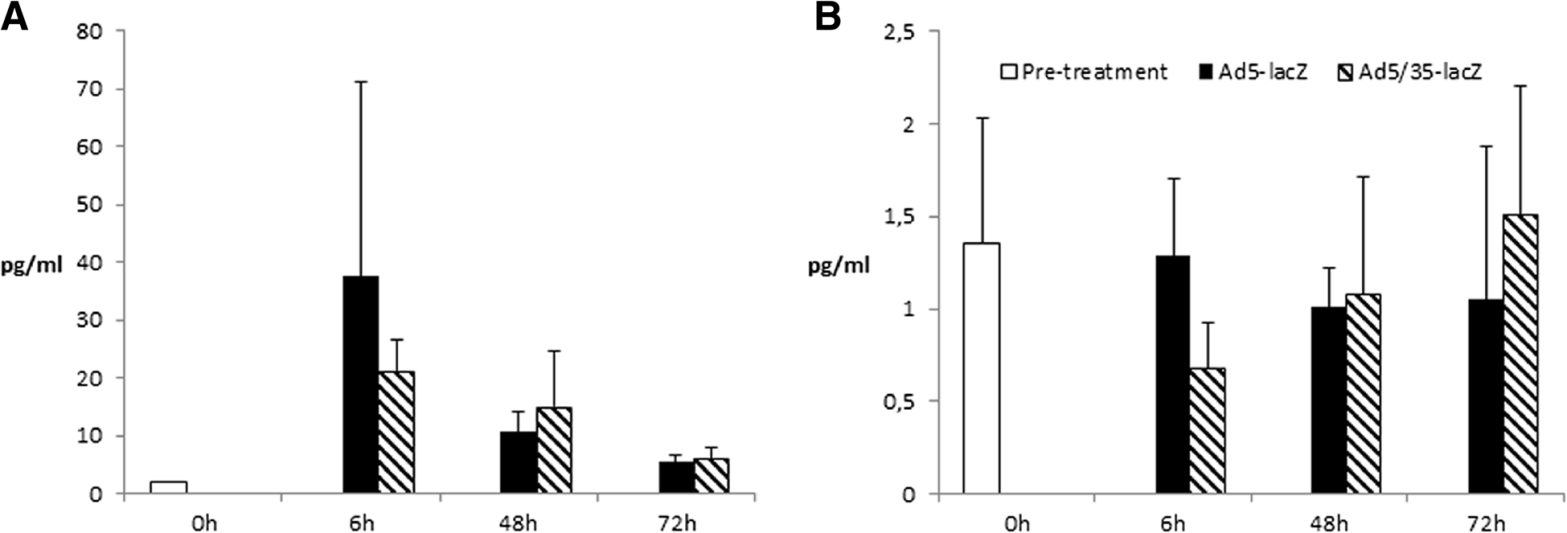
**Analysis of serum cytokine levels from 0 to 3 days post injection.** No statistically significant elevation of serum cytokine levels were observed for **A**) IL-6 or **B**) TNF-α. Although both viruses raised IL-6 levels within six hours post injection, after 3 days this elevation was observed to be again close to normal level. TNF-α levels did not show similar transient change.

In order to analyze systemic toxicity, blood serum IL-6 and TNF-α levels were analyzed at 6, 48 and 72 hours post injection (Figure 5). No significant elevation or differences between Ad5 and Ad5/35 were observed for either cytokine. Intramyocardial injection of both viruses raised IL-6 levels within six hours post injection, but after 3 days this elevation was observed to be again close to pre injection levels. TNF-α levels did not show similar transient change. We also evaluated the presence of Ad DNA in the lung, liver and spleen six days post injection by qualitative PCR. No adenoviral DNA was detected in any of the tested tissues of either Ad5-lacZ or Ad5/35-lacZ treated mice (data not shown).

## Discussion

Despite the availability of several promising genes to treat cardiovascular disease, their optimal delivery to the heart remains a challenge. A number of gene delivery vectors and methods of administration have previously been used for cardiovascular applications with variable success [9]. In this paper we combined two promising approaches, hybrid adenoviral vectors and ultrasound-guided intracardiac delivery, and analyzed the differences in transduction efficiency and toxicity after intracardiac injection between Ad5 and Ad5/35 vectors. We used a transgenic mouse strain hCD46Ge, which expresses the Ad5/35 receptor, human CD46, in a similar pattern as in humans, to compare the viruses in a situation comparable to man [21,25].

The viral vectors were injected to the left ventricle wall by using a closed-chest ultrasound guided system. With this system vectors can be administered directly into the left ventricle wall, without traumatic open-chest surgery. The injection leaves only a minor needle track and no other effects to cardiac function were observed. Safety of this injection method has been shown in healthy C57BL mice by advancing the needle 1-5 times to myocardium and also by repeated saline injections without compromising left ventricular function (unpublished data, Koskenvuo).

Transductional targeting has been widely used to improve efficacy and reduce toxicity in various gene therapy applications, most notably in cancer gene therapy [26]. Numerous reports have demonstrated marked improvements in therapeutic efficacy with the use of the hybrid adenovirus technology. For cardiovascular gene therapy, however, reports on transductional targeting applications for the myocardium have thus far been limited. We analyze here for the first time the effect of adenoviral transductional targeting on transgene expression and toxicity after intramyocardial closed-chest injection. Most previous studies involving adenoviral activity in the heart are biodistribution studies after intravenous (i.v.) administration. It has been shown previously that after systemic i.v. administration both Ad5-lacZ and Ad5/35-lacZ genomes are present at relatively high concentrations in the hearts of hCD46Ge mice as well as baboons and for Ad5/35-lacZ heart is one of the most effectively transduced tissues in both models [27,28]. Interestingly, with the primate model no transgene activity was reported in the heart tissue for any vector despite the presence of viral genomes. Analysis of genome copies present in tissues does not discriminate between active or inactive viral particles or between intracellular genomes and viruses in extracellular space. Unfortunately, only this method of analysis was employed by Ganesh *et al.* (2009). Here we show also the expression and activity of viral transgene in hCD46Ge mice, indicating that transgenes from both vectors are transcribed in our murine model. In their work Ganesh *et al.* (2009) also analyzed vector-mediated toxicity by measuring serum IL-6 and liver enzyme levels and showed decreased overall toxicity with Ad5/35-lacZ as compared to Ad5 vector after i.v. administration. The highest serum IL-6 levels in our experiment were 87 pg/ml, only 9% of the reported serum IL-6 levels after i.v. administration (Figure 4) [27]. In addition, our analysis of organs other than the heart revealed no signs of toxicity nor adenoviral DNA, indicating that systemic toxicity is low in our intracardiac injection model. Other vector systems have previously been used for gene transfer into the heart, including adeno-associated virus (AAV). Previous studies have demonstrated high tropism for the myocardium for AAV serotypes 1,6,8 and 9, making the AAV system a potentially attractive delivery vehicle for cardiovascular gene therapy [29]. The advantages of adenovirus-based approach include higher payload capacity and long experience from clinical trials, and serotype-modification strategies can potentially alleviate the strong immune response associated with adenovirus treatment.

There are also a few previous reports using ultrasound-guided intramyocardial injection of adenovirus in mice. Huusko *et al.* (2010) recently used this method to inject Ad5 expressing lacZ or different isoforms of VEGF, and the inflammatory reaction was found to be moderate at 14 days post injection, and the level of tissue damage was found to be also dependent on the transgene with VEGF-A giving rise to more inflammatory reaction than lacZ [30]. Li *et al.* (2005) used an Ad5 vector with deletions of the E1, E2a, and E3 regions expressing inducible nitric oxide synthase and reported little inflammatory reaction after intramyocardial delivery [31].

Our previous studies on adenoviral receptor expression in human heart tissue suggested that the native Ad5 capsid configuration would be better suited for cardiac gene transfer than Ad5/35 vectors as CAR had higher levels of expression than CD46 in both normal and dilated cardiomyopathy hearts [19]. As expected, the injection of unmodified Ad5 vector resulted in significantly more efficient transgene expression than administration of hybrid vector Ad5/35 (Figure 3, Figure 4). However, although both vectors elicited an immune response, this response was markedly more severe after Ad5 administration. At 6 days post injection there was not a significant difference in toxicities between the two viruses but by 14 days the Ad5 immune reaction showed signs of irreversible tissue damage with large areas of myocyte necrosis, whereas Ad5/35 had features of resolving immune reaction. These results are in accordance with a previous report from Nanda *et al.,* where the addition of Ad5 fiber knob to Ad35-based vectors was found to substantially increase the immunogenicity of Ad vectors [32]. LacZ transgene is also immunogenic and it is theoretically possible that the increased toxicity of Ad5 observed here could be caused by higher expression of lacZ. However, previous studies using different AAV vectors to deliver lacZ to mouse hearts have shown a mild immune response and no overt abnormalities in cardiac function despite high levels of lacZ expression, suggesting that immune response to lacZ does not play a significant role in our model [33].

The results suggest that, although Ad5 is more effective in transducing cardiac cells after intracardiac injection, this vector clearly has a worse safety profile and safer gene delivery can be achieved by using Ad5/35 hybrid vector. The definitive tissue damage after Ad5 injection will undermine the therapeutic effect of the delivered transgene and probably lead to severe side effects, although no adverse effects were evident during the 14 day post injection observation period. It must be also noted that even though Ad5 injection resulted in more efficient transgene expression, the cardiac tissue was not refractory to Ad5/35 infection and robust transgene expression was also achieved using this vector. It is notable that due to differences in viral particle/pfu ratios between the two viruses, Ad5/35-lacZ mice received 6.65 times more viral particles and still demonstrated less toxicity than A5-lacZ mice.

Results described here together with previous studies suggest that changing Ad5 fiber to those of Ad35 increases the safety profile of gene therapy vectors, although at the cost of some transduction efficiency. Ultrasound-guided intracardiac injection is a promising method for introducing genes to the heart. The increased safety with Ad5/35 vector warrants for further development of hybrid vector system for therapeutic purposes.

## Competing interests

The authors declare that they have no competing interests.

## Authors’ contribution

RT planned and performed the experiments, analyzed the data and wrote the manuscript. JK performed intracardial injections and reviewed the manuscript. MM performed intracardial injections and reviewed the manuscript. MSö analyzed the histology and reviewed the manuscript. SY planned the experiments and reviewed the manuscript. MSa planned the experiment, analyzed the data and wrote the manuscript. All authors read and approved the final manuscript.

## Supplementary Material

Additional file 1 **Figure S1.** Validation of ultrasound-guided intramyocardial injection technique for therapeutic purposes using methylene blue as an indicator for successful procedure. Left ventricle is cut to five short-axis sections from basis to apex (left to right in Figure). Dye injection produces darker outlook in the anterior myocardium of the three midventricular slices. Click here for file

## References

[B1] Gaggar A Shayakhmetov DM Lieber A CD46 is a cellular receptor for group B adenoviruses Nat Med 2003 9 11 1408 1412 10.1038/nm952 14566335

[B2] Bergelson JM Cunningham JA Droguett G Kurt-Jones EA Krithivas A Hong JS Horwitz MS Crowell RL Finberg RW Isolation of a common receptor for Coxsackie B viruses and adenoviruses 2 and 5 Science 1997 275 5304 1320 1323 10.1126/science.275.5304.1320 9036860

[B3] Tomko RP Xu R Philipson L HCAR and MCAR: the human and mouse cellular receptors for subgroup C adenoviruses and group B coxsackieviruses Proc Natl Acad Sci USA 1997 94 7 3352 3356 10.1073/pnas.94.7.3352 9096397 PMC20373

[B4] Roelvink PW Lizonova A Lee JG Li Y Bergelson JM Finberg RW Brough DE Kovesdi I Wickham TJ The coxsackievirus-adenovirus receptor protein can function as a cellular attachment protein for adenovirus serotypes from subgroups A, C, D, E, and F J Virol 1998 72 10 7909 7915 9733828 10.1128/jvi.72.10.7909-7915.1998 PMC110119

[B5] Sirena D Lilienfeld B Eisenhut M Kalin S Boucke K Beerli RR Vogt L Ruedl C Bachmann MF Greber UF Hemmi S The human membrane cofactor CD46 is a receptor for species B adenovirus serotype 3 J Virol 2004 78 9 4454 4462 10.1128/JVI.78.9.4454-4462.2004 15078926 PMC387694

[B6] Marttila M Persson D Gustafsson D Liszewski MK Atkinson JP Wadell G Arnberg N CD46 is a cellular receptor for all species B adenoviruses except types 3 and 7 J Virol 2005 79 22 14429 14436 10.1128/JVI.79.22.14429-14436.2005 16254377 PMC1280233

[B7] Seya T Hirano A Matsumoto M Nomura M Ueda S Human membrane cofactor protein (MCP, CD46): multiple isoforms and functions Int J Biochem Cell Biol 1999 31 11 1255 1260 10.1016/S1357-2725(99)00092-8 10605818

[B8] Wang H Li ZY Liu Y Persson J Beyer I Moller T Koyuncu D Drescher MR Strauss R Zhang XB Wahl JK 3rd Urban N Drescher C Hemminki A Fender P Lieber A Desmoglein 2 is a receptor for adenovirus serotypes 3, 7, 11 and 14 Nat Med 2011 17 1 96 104 10.1038/nm.2270 21151137 PMC3074512

[B9] Rissanen TT Yla-Herttuala S Current status of cardiovascular gene therapy Mol Ther 2007 15 7 1233 1247 10.1038/sj.mt.6300175 17505481

[B10] Campos SK Barry MA Current advances and future challenges in Adenoviral vector biology and targeting Curr Gene Ther 2007 7 3 189 204 10.2174/156652307780859062 17584037 PMC2244792

[B11] Gall J Kass-Eisler A Leinwand L Falck-Pedersen E Adenovirus type 5 and 7 capsid chimera: fiber replacement alters receptor tropism without affecting primary immune neutralization epitopes J Virol 1996 70 4 2116 2123 8642632 10.1128/jvi.70.4.2116-2123.1996 PMC190048

[B12] Amin KM Ad5 and Ad3 chimeric fiber travels into the cell without the CAR Cancer Biol Ther 2003 2 5 516 517 14614317 10.4161/cbt.2.5.520

[B13] Kanerva A Mikheeva GV Krasnykh V Coolidge CJ Lam JT Mahasreshti PJ Barker SD Straughn M Barnes MN Alvarez RD Hemminki A Curiel DT Targeting adenovirus to the serotype 3 receptor increases gene transfer efficiency to ovarian cancer cells Clin Cancer Res 2002 8 1 275 280 11801569

[B14] Segerman A Mei YF Wadell G Adenovirus types 11p and 35p show high binding efficiencies for committed hematopoietic cell lines and are infective to these cell lines J Virol 2000 74 3 1457 1467 10.1128/JVI.74.3.1457-1467.2000 10627557 PMC111481

[B15] Shayakhmetov DM Li ZY Ni S Lieber A Targeting of adenovirus vectors to tumor cells does not enable efficient transduction of breast cancer metastases Cancer Res 2002 62 4 1063 1068 11861383

[B16] Shinozaki K Suominen E Carrick F Sauter B Kahari VM Lieber A Woo SL Savontaus M Efficient infection of tumor endothelial cells by a capsid-modified adenovirus Gene Ther 2006 13 1 52 59 10.1038/sj.gt.3302598 16107861

[B17] Sova P Ren XW Ni S Bernt KM Mi J Kiviat N Lieber A A tumor-targeted and conditionally replicating oncolytic adenovirus vector expressing TRAIL for treatment of liver metastases Mol Ther 2004 9 4 496 509 10.1016/j.ymthe.2003.12.008 15093180

[B18] Suominen E Toivonen R Grenman R Savontaus M Head and neck cancer cells are efficiently infected by Ad5/35 hybrid virus J Gene Med 2006 8 10 1223 1231 10.1002/jgm.957 16941521

[B19] Toivonen R Mayranpaa MI Kovanen PT Savontaus M Dilated cardiomyopathy alters the expression patterns of CAR and other adenoviral receptors in human heart Histochem Cell Biol 2010 133 3 349 357 10.1007/s00418-009-0666-1 19957088

[B20] Mrkic B Pavlovic J Rulicke T Volpe P Buchholz CJ Hourcade D Atkinson JP Aguzzi A Cattaneo R Measles virus spread and pathogenesis in genetically modified mice J Virol 1998 72 9 7420 7427 9696838 10.1128/jvi.72.9.7420-7427.1998 PMC109970

[B21] Kemper C Leung M Stephensen CB Pinkert CA Liszewski MK Cattaneo R Atkinson JP Membrane cofactor protein (MCP; CD46) expression in transgenic mice Clin Exp Immunol 2001 124 2 180 189 10.1046/j.1365-2249.2001.01458.x 11422193 PMC1906059

[B22] Bautista DS Hitt M McGrory J Graham FL Isolation and characterization of insertion mutants in E1A of adenovirus type 5 Virology 1991 182 2 578 596 10.1016/0042-6822(91)90599-7 1827228

[B23] Shayakhmetov DM Papayannopoulou T Stamatoyannopoulos G Lieber A Efficient gene transfer into human CD34(+) cells by a retargeted adenovirus vector J Virol 2000 74 6 2567 2583 10.1128/JVI.74.6.2567-2583.2000 10684271 PMC111745

[B24] Springer ML Sievers RE Viswanathan MN Yee MS Foster E Grossman W Yeghiazarians Y Closed-chest cell injections into mouse myocardium guided by high-resolution echocardiography Am J Physiol Heart Circ Physiol 2005 289 3 H1307 H1314 10.1152/ajpheart.00164.2005 15908468

[B25] Johnstone RW Loveland BE McKenzie IF Identification and quantification of complement regulator CD46 on normal human tissues Immunology 1993 79 3 341 347 8406563 PMC1421998

[B26] Bachtarzi H Stevenson M Fisher K Cancer gene therapy with targeted adenoviruses Expert Opin Drug Deliv 2008 5 11 1231 1240 10.1517/17425240802507636 18976133

[B27] Ganesh S Gonzalez-Edick M Gibbons D Waugh J Van Roey M Jooss K Evaluation of biodistribution of a fiber-chimeric, conditionally replication-competent (oncolytic) adenovirus in CD46 receptor transgenic mice Hum Gene Ther 2009 20 10 1201 1213 10.1089/hum.2009.045 19572803

[B28] Ni S Bernt K Gaggar A Li ZY Kiem HP Lieber A Evaluation of biodistribution and safety of adenovirus vectors containing group B fibers after intravenous injection into baboons Hum Gene Ther 2005 16 6 664 677 10.1089/hum.2005.16.664 15960598 PMC1351080

[B29] Lyon AR Sato M Hajjar RJ Samulski RJ Harding SE Gene therapy: targeting the myocardium Heart 2008 94 1 89 99 10.1136/hrt.2007.116483 18083952

[B30] Huusko J Merentie M Dijkstra MH Ryhanen MM Karvinen H Rissanen TT Vanwildemeersch M Hedman M Lipponen J Heinonen SE Eriksson U Shibuya M Yla-Herttuala S The effects of VEGF-R1 and VEGF-R2 ligands on angiogenic responses and left ventricular function in mice Cardiovasc Res 2010 86 1 122 130 10.1093/cvr/cvp382 19955220

[B31] Li Q Guo Y Tan W Stein AB Dawn B Wu WJ Zhu X Lu X Xu X Siddiqui T Tiwari S Bolli R Gene therapy with iNOS provides long-term protection against myocardial infarction without adverse functional consequences Am J Physiol Heart Circ Physiol 2006 290 2 H584 H589 16172153 10.1152/ajpheart.00855.2005 PMC3648984

[B32] Nanda A Lynch DM Goudsmit J Lemckert AA Ewald BA Sumida SM Truitt DM Abbink P Kishko MG Gorgone DA Lifton MA Shen L Carville A Mansfield KG Havenga MJ Barouch DH Immunogenicity of recombinant fiber-chimeric adenovirus serotype 35 vector-based vaccines in mice and rhesus monkeys J Virol 2005 79 22 14161 14168 10.1128/JVI.79.22.14161-14168.2005 16254351 PMC1280229

[B33] Bish LT Morine K Sleeper MM Sanmiguel J Wu D Gao G Wilson JM Sweeney HL Adeno-associated virus (AAV) serotype 9 provides global cardiac gene transfer superior to AAV1, AAV6, AAV7, and AAV8 in the mouse and rat Hum Gene Ther 2008 19 12 1359 1368 10.1089/hum.2008.123 18795839 PMC2940566

