# Supplementary figures and images for: Intracardiac injection of a capsid-modified Ad5/35 results in decreased heart toxicity when compared to standard Ad5
Source: Virol J. 2012 Nov 29;9:296. doi: 10.1186/1743-422X-9-296 (PMC3546865; doi:10.1186/1743-422X-9-296)

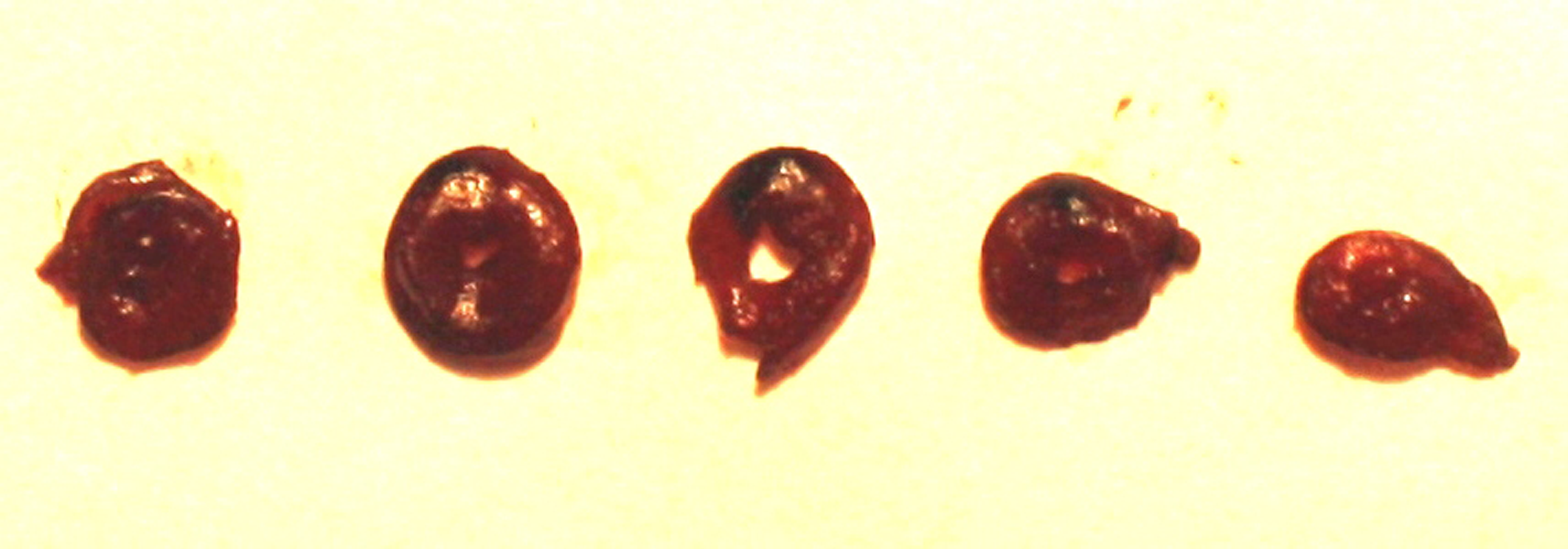

Supplement: Additional file 1 — Figure S1. Validation of ultrasound-guided intramyocardial injection technique for therapeutic purposes using methylene blue as an indicator for successful procedure. Left ventricle is cut to five short-axis sections from basis to apex (left to right in Figure). Dye injection produces darker outlook in the anterior myocardium of the three midventricular slices. [file 1743-422X-9-296-S1.tiff]
